# Supplementation of a β-mannanase enzyme reduces post-weaning diarrhea and antibiotic use in piglets on an alternative diet with additional soybean meal

**DOI:** 10.1186/s40813-021-00191-5

**Published:** 2021-01-11

**Authors:** Frédéric Vangroenweghe, Karl Poulsen, Olivier Thas

**Affiliations:** 1 Elanco, BU Food Animals, Elanco Benelux, Plantijn en Moretuslei 1 – 3rd floor, 2018 Antwerpen, Belgium; 2 Faculty of Veterinary Medicine, Unit of Porcine Health Management, Ghent University, Merelbeke, Belgium; 3 Elanco, BU Nutritional Health, Plantijn en Moretuslei 1 – 3rd floor, 2018 Antwerpen, Belgium; 4 I-BioStat, Data Science Institute, Hasselt University, Campus Diepenbeek, Agoralaan gebouw D, 3590 Diepenbeek, Belgium; 5 Department of Data Analysis and Mathematical Modelling, Faculty of Bioscience Engineering, Ghent University, Coupure Links 653, 9000 Ghent, Belgium; 6 National Institute of Applied Statistics Research Australia (NIASRA), University of Wollongong, Northfields Ave, Wollongong, NSW 2522 Australia

**Keywords:** β-Mannanase, Protein substitution, Weaned piglets, Performance, Antibiotic reduction

## Abstract

Enzyme supplementation with a β-mannanase to degrade β-mannan fibers present in the diet has been shown to restore and improve performance in swine. The current study was conducted on a farm which had historical episodes of post-weaning diarrhea. In total, 896 newly weaned piglets were enrolled in two consecutive trials. Each trial consisted of 32 pens of 14 piglets housed in one large post-weaning compartment. Piglets at the same feeder were randomly assigned to the two treatment groups. The study compared the performance of post-weaned piglets fed either a commercial 3-phase nursery diet (Control) or an adapted diet supplemented with a β-mannanase (Hemicell HT; Elanco) (Enzyme), with some of the more expensive proteins replaced by soy bean meal in phase 1 and 2, and net energy (NE) content reduced by 65 kcal/kg in phase 3. All data analyses were performed using R version 3.6.3 (R Core Team, 2020). All tests were performed at the 5% level of significance. When multiple testing was involved, the nominal 5% Familywise Error Rate (FWER) was used. The study showed similar performance on the alternative diet with β-mannanase and the common commercial diets (*P* >  0.05). However, the Enzyme treated group had a significantly better general clinical score. Moreover, the number of individual treatments was a factor *exp*(0.69441) or 2 (CI 95% [1.46; 2.74]) higher (*P* < 0.001) in the Control group as compared to the Enzyme treated group. The number of treated animals was a factor *exp*(0.62861) or 1.87 (CI 95% [1.43; 2.53]) higher (*P* < 0.001) and the number of pigs with a repeated treatment was a factor *exp*(0.9293) or 2.53 (CI 95% [1.26; 5.09]) higher (*P* = 0.009) in the Control group as compared to the Enzyme treated group. In total, 7 (1.56%) piglets died in the Control group, whereas only 2 (0.45%) piglets died in the Enzyme treated group. The hazard ratio for mortality in the Control group relative to the Enzyme treated group was and estimated as 1.74 (CI 95% [0.51; 5.96]). Thus, the Control group had a non-significantly (*P* = 0.375) increased mortality. In conclusion, the results suggest that the use of an exogenous heat-tolerant β-mannanase allowed reduced levels of expensive protein sources to be used in the first two diets fed post-weaning, and 65 kcal/kg lower net energy content to be used in the third diet without adverse effects on intestinal health or overall performance. In fact, the occurrence of PWD and number of individual treatments during the post-weaning period were significantly reduced on the β-mannanase supplemented diets.

## Introduction

Piglet post-weaning diets are by far the most expensive diets in the swine industry, mainly due to the need to include highly digestible feed ingredients to reduce post-weaning diarrhea (PWD) and to optimize growth performance. Globally, soybean meal (SBM) is by far the most widely used protein source in animal feeds, accounting for nearly 69% of all protein sources [1]. Soybean meals’ widespread use is motivated by its high content of digestible protein, good amino acid balance and relatively low cost. It would therefore be economically advantageous, if some of the expensive protein sources that are generally considered necessary in diets for newly weaned piglets could be substituted with SBM. Unfortunately, SBM contains 17 to 27% non-starch polysaccharides (NSP), which are indigestible for monogastric animals [2], together with several antinutritive factors, such as trypsin inhibitors, antigenic factors, and phytate [1]. β-Mannan is another antinutritive factor found in SBM and many other common feed ingredients [3], which has received increasing attention in recent years. β-Mannans are linear polysaccharides composed of repeating units of β-1,4-mannose and α-1,6-galactose and/or glucose units attached to the β-mannan backbone [4, 5]. They are considered unsuitable for young piglets due to their antinutritive properties, mainly due to the stimulation of the innate immune response. The innate immune cells identify pathogens using distinct molecules, called pathogen associated molecular patterns (PAMP), expressed on the pathogen surface [6]. Binding of PAMP to pathogen recognition receptors (PRR) present on innate immune cells, result in the release of innate defense molecules such as reactive oxygen and nitrogen species, bacteriolytic enzymes, antimicrobial peptides and complement proteins [7]. These PAMP include complex polysaccharides such as β-mannan [6]. Therefore, β-mannans from feed can create a false signal about the presence of pathogens in the gut, that elicits an unwarranted immune activation [8, 9], which is also known as a feed-induced immune response (FIIR [10];). This recognition mistake leads to a futile immune response that causes energy and nutrients to be wasted [4]. Hydrolysis of these β-mannans through inclusion of exogenous β-mannanase enzymes can reduce and potentially eliminate their ability to induce FIIR.

In poultry, the inclusion of dietary β-mannanase has been shown to improve daily gain and feed efficiency, while decreasing digesta viscosity [11], and to upregulate a broad range of metabolic functions related to digestion, metabolism and immunity [10]. Moreover, the beneficial effects of β-mannanase addition in chickens, challenged with *Eimeria* sp. and *Clostridium perfringens*, were observed with improved performance and reduced lesion scores in disease-challenged animals [12].

Supplementation of β-mannanase to low- and high-mannan diets has the potential to improve the performance of growing pigs [13]. Moreover, palm kernel meal (PKM) or copra meal may partially replace corn and SBM without reducing pig performance if β-mannanase is supplemented to the diet [13, 14]. The improved overall pig performance following supplementation of β-mannanase to corn-SBM-PKM diets might be due to increased adjusted ileal digestibility of different amino acids [15–17]. Others concluded that β-mannanase improved growth performance in both weanling and growing-finishing pigs on corn-SBM diets [18–20] with minimal effects on nutrient digestibility [19].

Additionally, β-mannanase supplementation to corn-SBM diets reduced the population of fecal coliforms and tended to reduce the NH_3_ concentration of fecal slurry after 24 h fermentation [21]. The reduction of fecal coliforms might impact the environmental infection pressure of coliforms, related to clinical problems of PWD. Therefore, inclusion of β-mannanase in corn-SBM diets of weanling piglets might be beneficial to reduce gut health problems in these younger animals [21]. Another study demonstrated in vivo anti-inflammatory activity of mannanase-hydrolyzed copra meal in a porcine colitis model, with decreased expression of mRNA for ileal IL-1β, IL-6, IL-17 and TNF-α [22]. Indeed, LPS immune stimulation increased energy partitioning to the immune system by 23%, which impacted lipid deposition and weight gain in challenged young weaned piglets [23]. Innate immune activation is accompanied by down-regulation of anabolic functions [24], which translates into a reduced performance capacity. Understanding energy and nutrient partitioning in immune-stressed piglets may provide more insights into the effects of FIIR activation by β-mannans from feed.

The objective of the current study was to evaluate the effects of β-mannanase supplementation to nursery diets with reduced content of expensive, high quality proteins on performance and health of nursery piglets vaccinated against a natural *E. coli* PWD infection.

## Materials and methods

### Description of experimental farm

The field trial was performed on a conventional farrow-to-finish pig farm with 600 DanBred sows in Flanders (Belgium), managed in a 4-week batch-management system with 120 sows per production batch. The farm was managed on all-in/all-out basis in all production phases. This management approach improved the health status for several respiratory pathogens [25].

Piglets (DanBred x German Piétrain) were weaned at 21 days of age and housed in a specifically equipped post-weaning facility, where they were raised for 7 weeks (49 days). The post-weaning facility was equipped with 32 pens of 14 piglets, allocated in 8 rows of 4 pens, separated by 4 inspection aisles. Every two pens were equipped with a dry feeder with a waterer on each side, located at the pen partition, so each feeder fed 28 piglets. The pens were equipped with fully slatted plastic floors. Heating was provided by hot water tubes on the ceiling near the air inlet, and ventilation was performed through 3 ventilation tubes and fresh air entered through a perforated ceiling air inlet system.

### Health status and relevant vaccination schedules

The trial farm had a conventional health status, which meant it was negative for Pseudo Rabies Virus (PRV), Classical Swine Fever (CSF) and African Swine Fever (ASF) and positive for Porcine Reproductive and Respiratory Syndrome virus (PRRSV), Porcine Circovirus type 2 (PCV-2), *Mycoplasma hyopneumoniae* (*M. hyo*), atrophic rhinitis (*Pasteurella multocida* & *Bordetella bronchiseptica*).

Sows were vaccinated with several vaccines: PRRSV vaccination on day 60 in gestation with a live avirulent vaccine and 6 weeks prior to parturition, PCV-2, *E. coli + Clostridium*, atrophic rhinitis and *Glässerella parasuis*. Additional to these standard vaccines, all gilts were vaccinated against *M. hyo*, Influenza-A Virus - Swine (IAV-S), Parvovirus and *Erysipelothrix rhusiopathiae* during their 6-week quarantine and adaptation period. Piglets were vaccinated during the suckling period against *M. hyo* (14 days), and *E. coli* F4/F18 (18 days) and 7 days post-weaning (dpw) against PCV-2.

### Description of clinical problems of post-weaning diarrhea

The farm had a history of post-weaning diarrhea (PWD), recurrently diagnosed as enterotoxigenic *E. coli* (ETEC) F4, which expressed enterotoxins STa, STb and LT. Clinical signs of PWD were characterized by watery yellowish diarrhea from 3 dpw onwards and was controlled through preventive vaccination with a live, avirulent *E. coli* F4 and F18 vaccine (Coliprotec® F4/F18; Elanco) at 5 days pre-weaning. Since onset of immunity by this vaccine is 7 days, the piglets were vaccinated timely in relation to the onset of clinical signs of PWD. Without *E. coli* vaccination, at least 10 days of antimicrobial treatment would be necessary and mortality would rise up to 4–5%, as observed in previous trials with a non-vaccinated control group [26, 27]. The *E. coli* vaccination had been administered as standard for several years prior to the trial. The challenging F4-ETEC infection pressure on this farm combined with the *E. coli* F4/F18 vaccine has resulted in a stable clinical situation related to PWD, although F4-ETEC might still be diagnosed post-weaning. Since label claims of the *E. coli* vaccine state a reduction in F4-ETEC and F18-ETEC pathogen excretion following vaccination, it is not usual to diagnose F4-ETEC following vaccination. No other enteric pathogens were detected that might cause PWD.

### Experimental design

#### Treatment groups and feeding regimen

Two experimental treatments were used, where the Control group received the standard diets and Enzyme treated group received the adapted nursery diets. The inclusion of a negative control group with a 65 kcal/kg NE reduction and without Enzyme supplementation was sacrificed in order to obtain sufficient replicates for the required power of the study. A 3-phase feeding program with two basal diets was used, a common commercial feeding program, and a similar, adapted program with 300 g/tonne of a heat-tolerant endo-1,4-β-mannanase (Hemicell HT Dry; Elanco), where expensive protein sources were partially replaced by extruded SBM in phase 1 and fully replaced by dehulled SBM in phase 2, and the enzyme was formulated to provide 65 kcal/kg NE in phase 3. The enzyme was added to the mixer with other minor ingredients during production of the diets at the feed mill. The composition and nutrient content of the diets are given in Tables 1 and 2. Briefly, the diets used for the two treatments had similar nutrient content per kg in all three phases: digestible lysine content of 12.1 g/kg in phase 1, 11.8 g/kg in phase 2, and 11.8 g/kg in phase 3. Identical NE content of both Control and Enzyme treated group of 2440 kcal/kg in phase 1 and 2425 kcal/kg in phase 2 were used, while in phase 3, the NE content was 2399 kcal/kg in the Control diet and 2334 kcal/kg in Enzyme treated diet for a calculated NE reduction of 65 kcal/kg (Table 2).

**Table 1:** Composition of the diets for phase 1, 2 and 3 in both Control and Enzyme treated group

Composition, %	Weeks 1–2		Weeks 3–4		Weeks 5–7	
	Control	Enzyme	Control	Enzyme	Control	Enzyme
Barley 2018, cleaned	20.0	20.0	20.0	20.0	25.0	25.1
Wheat 2018, cleaned	18.8	19.5	24,6	28.8	16.5	18.2
Soybean meal, extruded (Danex)	10.4	11.3	8.5	8.5	–	–
Soybean meal 49%	2.5	2.5	10.4	12.4	18.9	18.7
Corn	5.0	5.0	15.0	10.0	15.0	15.0
Oat, rolled	5.0	5.0	–	–	–	–
Barley, rolled	5.0	5.0	–	–	–	–
Corn, extruded	5.0	5.0	–	–	–	–
Wheat shorts	–	–	4.0	4.0	8.3	7.9
Wheat feed	–	–	–	–	5.0	5.0
Vitamin/mineral premix^a^	3.0	3.0	3.0	3.0	3.0	3.0
Sugar, palatine (Beneocarb)	1.0	1.0	1.0	1.0	–	–
Beet pulp	1.0	1.0	1.0	1.0	1.0	1.0
Cocoa oil	0.500	0.500	0.5	0.5	–	–
Soya oil	–	–	1.80	2.20	1.70	0.55
Animal fat	–	–	–	–	3.50	3.50
Citric acid	0.500	0.500	–	–	–	–
Chalk	0.290	0.155	0.305	0.295	0.64	0.65
PX Cu 0.3%	–	0.110	–	–	–	–
Lysine 70%	1.055	1.245	0.940	0.960	0.750	0.755
L-Threonine	0.325	0.310	0.295	0.275	0.230	0.230
DL-Methionine 99%	0.320	0.340	0.290	0.295	0.225	0.225
L-Valine	0.219	0.296	0.119	0.124	0.035	0.035
L-Tryptophan	0.089	0.080	0.075	0.064	0.049	0.049
Base mix 20%^b^	20.0	–	8.0	–	–	–
Base mix 16%^c^	–	16.0	–	6.4	–	–
Potato protein concentrate	1.14^d^	–	0.46^d^	–		
Forcital (extruded soya product)	1.71^d^	1.00	0.69^d^	–	–	–
Wheat gluten	1.14^d^	1.14	0.46^d^	–	–	–
Hemicell HT Dry	–	0.030	–	0.030	–	0.030

^a^ The vitamin/mineral premix was calculated to provide per kg complete feed:150,000 IE vit A, 2000 IE/kg vit D_3_, 100 IE/kg vit E, 2.0 mg/kg vit K_3_, 2.0 mg/kg vit B_1_, 6.0 mg/kg vit B_2_, 15.0 mg/kg Ca D-pantothenate, 2.0 mg/kg vit B_6_, 30.0 μg/kg vit B_12_, 30.0 mg/kg niacinamide, 1.0 mg/kg folic acid, 100 μg/kg biotine, 150 mg/kg betainehydrochloride, 40 mg/kg vit C, 1.5 mg/kg potassium iodine, 0.42 mg/kg sodiumselenite, 20 mg/kg Cu-II-sulphate, 70 mg/kg Cu-II-chelate, 50 mg/kg manganese, 105 mg/kg ZnO, 100 mg/kg Fe_2_SO_4_, 152 U/kg 1,3(4)-β-glucanase (Axtra XB 201), 1220 U/kg 1,4-β-xylanase (Axtra XB 201), 1500 FTU/kg 6-phytase (Axtra PHY)

^b^ Base mix 20%: Supplied 25% lactose, 5.71% potato protein conc., 5.71% wheat gluten, and 8.57% Forcital (extruded soya)

^c^ Base mix 16%: As Base mix 20% without 20% protein products (potato protein concentrate, wheat gluten and Forcital)

^d^ Provided as part of base mix 20%. Shown for comparison to the levels in the diets with Hemicell

**Table 2:** Calculated nutrient content of the diets for phase 1, 2 and 3 in both Control and Enzyme treated group

Nutrient content	Weeks 1–2		Weeks 3–4		Weeks 5–7	
	Control	Enzyme	Control	Enzyme	Control	Enzyme
Dry matter, g/kg	889.1	889.5	882.7	883.1	882.8	881.0
Moisture, g/kg	110.9	110.5	117.3	116.9	117.2	118.7
Crude protein, g/kg	169.9	165.5	179.1	179.0	179.6	180.0
Crude fiber, g/kg	34.2	34.3	35.6	35.9	39.5	39.5
Crude fat, g/kg	48.3	49.4	60.7	63.1	74.8	62.4
Ash, g/kg	47.1	46.0	47.4	47.6	51.0	51.0
Sugars, g/kg	67.2	61.0	53.7	52.8	40.5	40.5
Lactose, g/kg	50.0	50.0	20.0	20.0	–	–
Starch, g/kg	344.9	367.0	358.0	359.7	351.7	360.9
Net energy, kcal/kg	2440	2439	2425	2426	2399	2334
Lysine, total, g/kg	13.74	13.62	13.47	13.43	12.38	12.39
Lysine, digestible, g/kg	12.10	12.10	11.81	11.82	10.80	10.81
Methionine, digestible, g/kg	5.19	5.21	4.96	4.95	4.31	4.32
Methionine+Cysteine, digestible, g/kg	7.37	7.89	7.23	7.43	6.64	6.66
Threonine, digestible, g/kg	7.60	7.64	7.43	7.44	6.82	6.82
Tryptophan, digestible, g/kg	2.42	2.42	2.36	2.36	2.16	2.16
Isoleucine, digestible, g/kg	5.09	5.08	5.43	5.68	5.40	5.40
Ca, g/kg	5.48	5.46	5.10	5.12	5.90	5.91
P, g/kg	5.21	5.25	5.60	5.63	5.91	5.90
P, digestible, g/kg	4.18	4.16	4.33	4.33	4.22	4.22
Na, g/kg	2.64	2.70	2.42	2.44	2.38	2.38
K, g/kg	7.23	7.19	8.10	8.41	8.79	8.77
Cl, g/kg	4.15	4.34	3.41	3.45	3.05	3.05
Cu, mg/kg	140	140	155	155	154	154
Zn (added), mg/kg	105	105	105	105	105	105

The 3-phase feeding programs was offered as following: piglets were fed phase 1 feeds from days 1–15, phase 2 feeds from days 16–27 and phase 3 diets from days 28–49.

Substitution of expensive protein sources and reduction of 65 kcal/kg NE resulted in a substantial reduction of the feed costs as shown in Table 3. The reduction of expensive protein sources in phase 1 had a substantial impact and reduced the feed cost by € 29.00 per tonne, whereas the substitutions in phase 2 and energy reduction in phase 3 had a smaller impact of € 6.77 and € 4.45 per tonne, respectively.

**Table 3:** Feed price (€/tonne) for both Control and Enzyme treated diets in phase 1, 2 and 3, and reduction (expressed as %) in feed price between Control and Enzyme treated diets

Feeding phase	Feed price (€/tonne)		Reduction in feed price Control vs. Enzyme (%)
	Control	Enzyme	
Phase 1	579.00	560.18	- 3.25
Phase 2	426.00	419.23	- 1.59
Phase 3	356.00	351.55	- 1.25

#### Study animals

Two batches of 448 newly weaned piglets were allocated to treatment by weight and sex. Castrated males and females were penned separately. The same number of castrated male and female pigs were allocated to both treatment groups. All piglets were ear tagged with individual identification numbers. Sixteen replicate pens of 14 piglets were included per treatment in each batch for a total of 32 replicates per treatment. The piglets at each feeder were randomly assigned to one of both treatment group: Control or Enzyme treated.

#### Performance data collection

Performance data were collected such as bodyweight at day 1 (trial start), each feed change and day 49 (end of trial). On days 1 through 11, piglet uniformity was evaluated and described by pen. Feed allocation was assumed to equal feed intake and recorded daily as feed bags of 25 kg were added to the feeders. Average daily weight gain (ADWG), average daily feed intake (ADFI), and feed conversion ratio (FCR) were calculated for each feeding period and overall (Table 4). No adjustments for mortality and culls were performed, since mortality was below 2.0% and no culls occurred during the trial.

**Table 4:** Average daily weight gain (ADWG; g/piglet/d; mean ± SEM), average daily feed intake (ADFI; g/piglet/d; mean ± SEM), feed conversion rate (FCR; kg feed/kg weight gain; mean ± SEM) in phase 1 (0–15 dpw), phase 2 (16–27 dpw), phase 3 (28–49 dpw) and overall (0–49 dpw). Piglets in the Control group were fed a standard diet, whereas piglets in the Enzyme treated group were fed an adapted diet with more soybean meal in phase 1 and 2, and a decreased level of 65 kcal/kg net energy in phase 3. No significant differences (*P* >  0.05) between groups were observed

Production Parameter	Production Phase	Control	Enzyme	Enzyme vs. Control (%)	***P***-value
*Average Daily Weight Gain (g/piglet/d; mean ± SEM)*					
	Phase-1	129.5 ± 3.5	117.1 ± 3.2	- 9.57	> 0.05
	Phase-2	324.6 ± 17.1	303.0 ± 18.2	- 6.65	> 0.05
	Phase-3	572.5 ± 48.0	566.7 ± 47.1	- 1.01	> 0.05
	Overall	342.4 ± 6.6	331.9 ± 8.9	- 3.24	> 0.05
*Average daily feed intake (g/piglet/d; mean ± SEM)*					
	Phase-1	153 ± 8	143 ± 7	- 6.53	> 0.05
	Phase-2	436 ± 25	409 ± 25	- 6.19	> 0.05
	Phase-3	1019 ± 65	982 ± 53	- 3.63	> 0.05
	Overall	536 ± 99	512 ± 95	- 4.47	> 0.05
*Feed conversion ratio (kg feed/kg gain; mean ± SEM)*					
	Phase-1	1.32 ± 0.025	1.36 ± 0.029	+ 3.03	> 0.05
	Phase-2	1.29 ± 0.017	1.29 ± 0.020	0.00	> 0.05
	Phase-3	1.76 ± 0.073	1.79 ± 0.067	+ 1.70	> 0.05
	Overall	1.46 ± 0.071	1.48 ± 0.070	+ 1.37	> 0.05

Mortality, scours, and veterinary treatments (with presumed diagnosis) were recorded daily (Tables 5 and 6). Dead piglets were not submitted to necropsy.

**Table 5:** Area under the curve (expressed in AUC_0–11_) of pen fecal clinical score and pen general clinical score (GCS), time to maximal FCS and GCS (expressed in dpw; mean ± SEM) for piglets during the first 11 dpw and mortality during the entire trial period (N piglets and %) are given. Piglets in the Control group were fed a standard diet, whereas piglets in the Enzyme treated group were fed an adapted diet with more soybean meal in phase 1 and 2, and a decrease level of 65 kcal/kg net energy in phase 3. Different superscript letters indicate statistically significant differences (*P* < 0.05)

	Control	Enzyme
Pen FCS^1^ (AUC_0–11_)	14.40 ± 0.215 ^a^	13.65 ± 0.134 ^a^
Time to maximal FCS (dpw)	7.06 ± 0.100 ^a^	6.25 ± 0.077 ^a^
Pen GCS^2^ (AUC_0–11_)	116.0 ± 0.23 ^a^	126.5 ± 0.14 ^b^
Time to minimal GCS (dpw)	6.78 ± 0.108 ^a^	6.85 ± 0.042 ^a^
Mortality, % (N)	1.79 (8) ^a^	0.45 (2) ^a^

^1^ Pen FCS was scored daily on a score from 0 (= normal) to 2 (= watery diarrhea)

^2^ GCS was scored daily on a score from 0 (= very bad) to 10 (= excellent)

*AUC*_*0–11*_, area under the curve 0–11 dpw, *dpw* Days post-weaning

**Table 6:** Antimicrobial treatments during the feed trial categorized by phase and reason for treatment, including an overall total and number of unique piglets treated and number of piglets receiving a repeated treatment with an antimicrobial during the trial. Piglets in the Control group were fed a standard diet, whereas piglets in the Enzyme treated group were fed an adapted diet with more soybean meal in phase 1 and 2, and a decrease level of 65 kcal/kg net energy in phase 3. Different superscript letters indicate statistically significant differences (*P* < 0.05)

Treatment	Control				Enzyme				***P***-value
Feeding phase	Ph-1	Ph-2	Ph-3	Overall	Ph-1	Ph-2	Ph-3	Overall	
**Diarrhea**	74	0	0	*74* ^*a*^	33	0	0	*33* ^*a*^	*P* = 0.282
**Small/unthrifty**	8	9	8	*25* ^*a*^	6	7	6	*19* ^*a*^	*P* = 1.000
**Leg infection**	3	8	5	*16* ^*a*^	1	2	3	*6* ^*a*^	*P* = 1.000
**Meningitis**	0	2	1	*3* ^*a*^	0	1	0	*1* ^*a*^	*P* = 0.283
**TOTAL**	85	19	14	*118* ^*a*^	40	10	9	*59* ^*b*^	*P* < 0.001
**N pigs treated (%)**	80	10	0	*90 (20.1)* ^*a*^	40	6	2	*48 (10.7)* ^*b*^	*P* < 0.001
**N pigs with repeated treatment (%)**	5	9	14	*28 (31.1)* ^*a*^	0	4	7	*11 (22.9)* ^*b*^	*P* = 0.009

#### Assessment of fecal clinical score and general clinical score

Pigs were evaluated daily and any unusual observations were recorded, including but not limited to altered behavior and disease. Diarrhea scores were assessed for each pen by scoring five droppings per pen based on the fecal clinical score (FCS) shown in Table 7 [26, 27]. The scoring was done by the same observer on days 0 to 11 post weaning and on day 49 (end of trial). The scoring was done by first counting all droppings with a score 2, then score 1 and finally score 0, until a total of 5 droppings had been recorded. Fecal clinical scores during the observation period were expressed as area under the curve (AUC_0–11_) and days to maximal FCS were calculated for each pen. Simultaneously, the uniformity and appearance of the piglets was scored by pen on a scale from 1 to 5, where score 1 represented poor appearance (heterogeneity, sunken flanks, long hair coat, deep eyes, …) and score 5 represents perfect condition (homogenous, filled belly, shiny pink skin, …). This general clinical score (GCS) was performed every day from weaning (0 dpw) until 11 dpw by the same observer. General clinical scores during the observation period were expressed as area under the curve (AUC_0–11_) and day to minimal GCS was calculated for each pen. Dead piglets were recorded by date, experimental group, pen and body weight. No necropsies were carried out.

**Table 7:** Comprehensive description of the pen fecal clinical score with its interpretation and the clinical aspect of the fecal clinical score (adapted from Vangroenweghe et al., 2020a [26]; Vangroenweghe et al., 2020b [27])

Score	Interpretation	Clinical aspect
0	Normal	Normal feces consistency
1	Pasty to mild	Soft pasty consistency with more particles than fluid
2	Moderate to severe	More fluid than particles

#### Veterinary treatments

Individual antibiotic treatments for PWD were performed as needed due to the critical state of the piglet and according to the clinical criteria of the farm veterinarian. The same veterinary products, active ingredients, formulations and dosages were used throughout the entire study period. Individual antibiotics treatments were recorded daily by date, product, dose, ID number of treated piglets, presumed cause of treatment, and number of times the treatment was repeated. For both treatment groups, the same antibiotic product and dosage was applied for the same indication.

Practically, diarrhea problems were treated using a combined product containing lincomycin-spectinomycin (Linco-Spectin; Zoetis, Louvain-la-Neuve, Belgium; 50 mg lincomycin + 100 mg spectinomycin per ml; 1 mL per 10 kg). Small, unthrifty piglets, piglets with leg problems and other pathologies were treated using a product containing amoxicillin (Duphamox LA 150 mg/ml; Zoetis, Louvain-la-Neuve, Belgium; 150 mg amoxicillin per ml; 1 ml per kg).

### Statistical analysis

All data analyses were performed using R version 3.6.3 [28]. All tests were performed at the 5% level of significance. When multiple testing was involved, the nominal 5% Familywise Error Rate (FWER) was used. For ADWG, ADFI and FCR, the average values were calculated within each trial, phase and treatment combination. These averages were considered as outcome variables and, within each phase, analyzed with two-way ANOVA models treatment and trial as factors (the latter is to be considered as a blocking variable. The *P-*values (one for each phase) for the F-test for the treatment effect were adjusted for multiple testing using the Bonferroni method. For weight, the average values were calculated within each trial and treatment combination. These averages were used as outcome variables and analyzes with two-way ANOVA models with trial and treatment as factors. Again, trial acted as a blocking factor. The F-test was used for testing the treatment effects. For FCS and GCS, the average values were calculated for each trial, pen, treatment and dpw combination. Subsequently, the average values of AUC_0–11_ for FCS and GCS were calculated for each trial and treatment combination, and they were used as outcome variables in two-way ANOVA models with with trial and treatment as factors. Trial acted again as a blocking factor. The F-test was used for testing the treatment effects. The treatment effect on the time to maximal FCS and the time to minimal GCS was testing with the Wilcoxon rank sum statistic, stratified according to trial, i.e. the test statistic is given by the sum of the two trial-specific Wilcoxon rank sum statistics. The null distribution was enumerated as a permutation null distribution, by (1000 times) randomly permuting the treatment labels within the trials (thus trial acted as a blocking factor, implying a randomization restriction). With this permutation null distributions, two-sided *p*-values were computed. The number of treated and the number of repeated was analyzed with a Poisson regression model, with Pen as a blocking factor. For each reason of treatment, the numbers in the two treatment groups were compared with a Pearson chi-squared test. The four *P*-values were adjected with the method of Bonferroni. The mortality, represented as time-to-death, was analyzed with a Cox proportional hazard model with treatment factor variables.

## Results

### Piglet weight and average daily weight gain

**Fig. 1:**
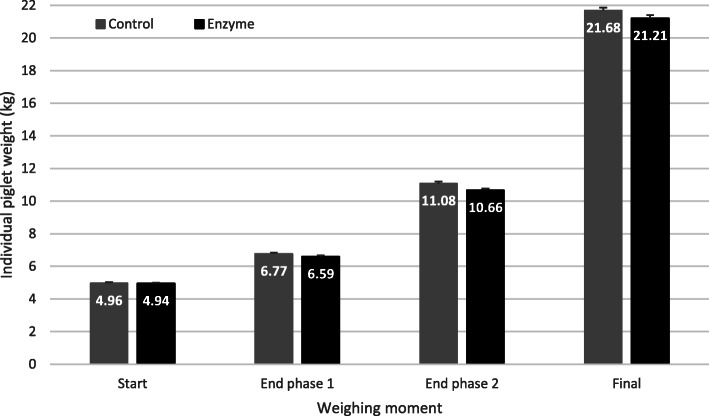
Individual piglet weight (kg; mean ± SEM) at weaning (Start; 0 dpw), first intermediate weighing (End phase 1; 15 dpw), second intermediate weighing (End phase 2; 27 dpw) and end of the trial (Final; 49 dpw). Piglets in the Control group were fed a standard diet, whereas piglets in the Enzyme treated group were fed an adapted diet with more soybean meal in phase 1 and 2, and a decrease level of 65 kcal/kg net energy in phase 3. No significant differences between groups were observed

Piglets were weaned at 21 days of age and an average weight of 4.95 kg (± 0.06) and randomly distributed into two treatment groups. No significant differences in piglet bodyweight were observed during the trial. However, on day 15, Control piglets were numerically heavier than the Enzyme treated piglets, 6.77 ± 0.073 kg vs. 6.59 ± 0.071 kg, respectively, on day 27 Control and Enzyme treated piglets weighed 11.08 kg ± 0.110 and 10.66 kg ± 0.106 respectively, and on the final weighting on day 49 they weighed 21.68 ± 0.177 kg vs. 21.21 ± 0.194 kg, respectively (Fig. 1).

Average daily weight gain in phase 1 was 12 g/day lower in the Enzyme treated piglets as compared to Control. In phase 2 and 3, the daily gain on the Enzyme treated piglets was numerically lower by 21 and 6 g/d respectively. Overall, the Enzyme treated piglets gained 10 g/day less than the Control piglets. None of the treatment differences in daily gain were significantly different (Table 4).

### Average daily feed intake and feed conversion rate

Similar ADFI for both treatments were observed in all three production phases. Piglets in the Control group tended to consume more feed than the Enzyme treated piglets. The ADFI in each of the phases differed by 10, 27 and 37 g/day, respectively. Overall, the Control piglets consumed 24 g/day more than the Enzyme treated piglets. However, none of the differences in ADFI were significant (*P* >  0.05) (Table 4).

No significant difference in feed conversion rate (FCR) were observed. In phase 1, FCR was numerically lower on the Control feed (1.32 ± 0.025) than on the Enzyme treated feed (1.36 ± 0.029). In phase 2, FCR was equal (1.29 ± 0.018) in both treatment groups, and in phase 3, FCR on the Enzyme treated feed was again numerically increased (1.79 ± 0.067) versus (1.76 ± 0.073). Overall, FCR on the Enzyme treated feed was slightly, though non-significantly higher (+ 1.37%) as compared to Control piglets (Table 4).

### Pen fecal clinical score and general clinical score

**Fig. 2:**
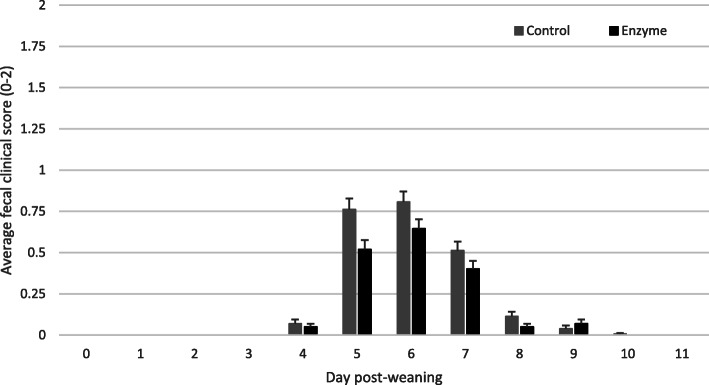
Average fecal clinical score (mean ± SEM) from 0 to 11 dpw. Five relevant fecal droppings were scored daily per pen during the observation period. Piglets in the Control group were fed a standard diet, whereas piglets in the Enzyme treated group were fed an adapted diet with more soybean meal in phase 1 and 2, and a decrease level of 65 kcal/kg net energy in phase 3. No significant differences between groups were observed

Pen FCS was collected daily for each individual pen from 0 to 11 dpw. Daily average pen FCS (mean ± SEM) is given in Fig. 2. Pen FCS, expressed as AUC_0–11_, was not significantly higher (*P* >  0.05) in the Control group as compared to the Enzyme treated group (Table 5). Although some numerical differences in time to maximal FCS, expressed as dpw, occurred between the treatments, no significant differences in the time to maximal FCS (*P* >  0.05) were observed (Table 5).

**Fig. 3:**
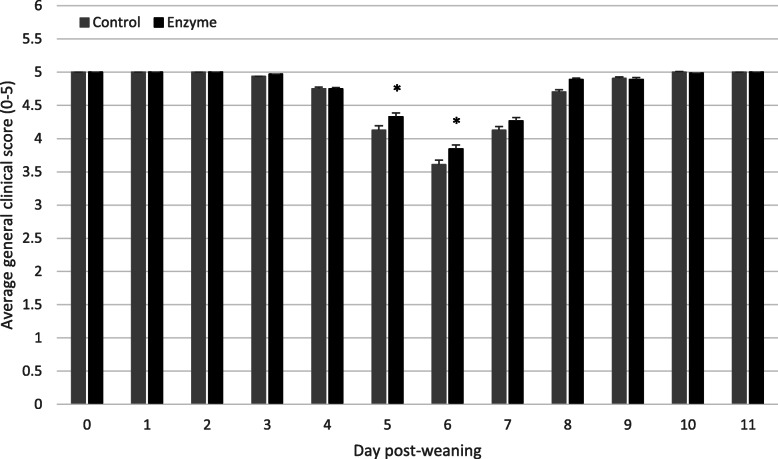
Average general clinical score (mean ± SEM) from 0 to 11 dpw. Piglets in each pen were scored (0–2) daily during the observation period. Piglets in the Control group were fed a standard diet, whereas piglets in the Enzyme treated group were fed an adapted diet with more soybean meal in phase 1 and 2, and a decrease level of 65 kcal/kg net energy in phase 3. Significant differences (*P* < 0.05) were indicated with an asterix (*)

Pen GCS was collected daily for each individual pen from 0 to 11 dpw. Daily average pen GCS (mean ± SEM) is given in Fig. 3. Pen GCS, expressed as AUC_0–11_, was significantly higher (*P* < 0.05) in the Enzyme treated group as compared to the Control group (Table 3). Although some numerical differences in time to minimal GCS, expressed as dpw, occurred between both treatment groups, no significant differences in the time to minimal GCS (*P* >  0.05) were observed (Table 5).

### Mortality

Data related to mortality are given in Table 5. In total, 7 (1.56%) piglets died in the Control group, whereas only 2 (0.45%) piglets died in the Enzyme treated group. In summary, the hazard ratio for mortality in the Control group relative to the Enzyme treated group was estimated as 1.74 (CI 95% [0.51; 5.96]). Although the piglets in the Control group had an increased risk of mortality, this effect was not significant (*P* = 0.375) (Table 5).

### Antimicrobial treatment

Antimicrobial treatment was registered per day and reason of treatment. The number of treatments administered for diarrhea tended to be higher (*P* >  0.05) in the Control group (*N* = 74) compared to the Enzyme treated group (*N* = 33) (Table 6). Moreover, all treatments against diarrhea were administered during phase 1 (day 1–14). Unthriftyness was the second most common reason for treatment, followed by leg infections (e.g. *S. suis* or *G. parasuis*). Only a few piglets suffered from meningitis due to *S. suis*. No significant differences (*P* >  0.05) in these treatments reasons were recorded between both treatment groups. In the Control group, the total number of antimicrobial treatments was a factor *exp*(0.69441) or 2 (CI 95% [1.46; 2.74]) higher as compared to the Enzyme treated group. Moreover, the total number of individually treated piglets was a factor *exp*(0.62861) or 1.87 (CI 95% [1.43; 2.53]) higher (*P* < 0.001) in the Control as compared to the Enzyme treated group. The number of piglets requiring repeated treatment differed (*P* = 0.009) between both treatments and was a factor *exp*(0.9293) or 2.53 (CI 95% [1.26; 5.09]) higher in the Control group as compared to the Enzyme treated group.

## Discussion

In the current study, we substituted a part of the most expensive protein sources (potato protein concentrate, Forcital (extruded soybean product) and wheat gluten) with Danex® (extruded SBM) and wheat in phase 1, and they were fully substituted with regular SBM in phase 2. Both soya products were expected to have a similar and relatively high β-mannan content, a known antinutritive factor [3], which may stimulate an innate immune response through their resemblance with PAMPs [6]. This activation has been called FIIR (Feed Induced Immune Response [10];) and leads to an unnecessary immune activation, causing energy and nutrients to be wasted [4]. Therefore, 300 g/tonne of an exogenous β-mannanase enzyme (Hemicell HT; Elanco, Greenfield, IA) was added to hydrolyze these antinutritive β-mannans in the trial feed. The results in phase 1 and phase 2 demonstrated no significant differences in the measured (piglet weight, ADFI) or calculated (ADWG, FCR) performance parameters between treatments. Although minor numerical differences were observed, the overall result confirmed that the addition of an exogenous β-mannanase to adapted formulations with more challenging, β-mannan-containing ingredients, allowed them to perform equally to the standard post-weaned diets. These results are in accordance with other recent studies in low- and high-mannan diets [13].

In phase 3, the formulation of the Control and Enzyme treated diets only differed in the inclusion of soya oil, which was reduced from 1.70 to 0.55% to obtain a net energy reduction of 65 kcal/kg feed. Again, in phase 3 only minor numerical performance differences were observed between treatments. The overall result confirmed that the addition of β-mannanase allowed performance to be maintained with diets formulated with reduced content of expensive protein sources in phase 1 and 2, and about 3% lower dietary energy content (65 kcal/kg net energy) in phase 3. In addition, a substantial economic advantage of using the enzyme could be calculated. Based on the feed prices presented in Table 3 and the actual feed intake, we obtained a 3 % reduction in the feed cost per produced piglet. Taking into account all costs (feed cost, veterinary treatment costs, mortality, basic piglet market price and supplements for weight above 20 kg), the income per produced piglet was € 0.58 higher for the Enzyme treated group. Others concluded that β-mannanase improved growth performance in both weanling and growing-finishing pigs on corn-SBM diets [18–20]. A diet with a 150 kcal/kg reduction in digestible energy supplemented with β-mannanase outperformed in weight gain and feed efficiency [18]. Others have also observed the energy sparing effect from supplementation of β-mannanase. For example the supplementation to a common nursery diet resulted in similar effects on performance of a comparable diet supplemented with 2% soya oil [19].

In poultry, beneficial effects of β-mannanase supplementation on the performance of chickens challenged with *Eimeria* sp. and *Clostridium perfringens* were observed together with reduced lesion scores in disease-challenged birds [12]. Therefore, we selected a field trial facility with a substantial intestinal challenge related to PWD due to *E. coli*, which was partly tackled using an oral live non-pathogenic *E. coli* vaccine (Coliprotec® F4/F18; Elanco, Greenfield, IA). In order to quantify intestinal and general health aspects, we recorded a fecal clinical score (FCS; adapted from [26, 27]) and a general clinical score (GCS; adapted from [26, 27]) during the most critical period post-weaning (from weaning until 11 dpw). Although some differences in kinetics could be observed for FCS, the treatment did not differ significantly their AUC and time to maximum FCS during the entire period from 0 to 11 dpw. In contrast, AUC of GCS was significantly better in the Enzyme treated group compared to the Control group, which means that the general condition, as scored by the farmer, was better in the Enzyme treated group. The time to minimal GCS, however, did not differ between treatments. Diseased piglets were only treated using single individual injections and whole group treatments were not administered at any time during the trial. All individual treatments were registered with date of treatment, reason for treatment and individual piglet identification. From Table 6, we can conclude that piglets in the Control group received significantly more treatments for diarrhea during phase 1 and significantly more treatments overall, resulting in more than double the number of injections as compared to the Enzyme treated group. From these data, it is also obvious that more than double the number of individual piglets were injected in the Control group and more than double (2.53) the number of piglets with a repeated treatment could be registered in the Control group as compared to the Enzyme treated group. Combining the treatment data with the GCS results, we can conclude that overall health status in the Enzyme treated group was significantly higher compared to the Control group. This observation is, to our knowledge, the first of its kind in pigs fed a corn-SBM diet supplemented with an exogenous β-mannanase, and confirms the earlier data on intestinal health in poultry following a challenge with *Eimeria* sp. and *Clostridium perfringens* [13].

## Conclusions

The current study results suggest that the use of an exogenous heat-tolerant β-mannanase allowed reduced levels of expensive protein sources to be used in the first two diets fed post weaning, and 65 kcal/kg lower net energy content to be used in the third without adverse effects on intestinal health or overall performance. In fact, the occurrence of post-weaning diarrhea and number of individual treatments during the post-weaning period were significantly reduced on the β-mannanase supplemented diets.

## Data Availability

The datasets analyzed during the current study are available from the corresponding author on reasonable request.

## Abbreviations

ADFI: Average daily feed intake
ADWG: Average daily weight gain
AUC: Area under the curve
dpw: Days post-weaning
ETEC: Enterotoxigenic *Escherichia coli*
FCR: Feed conversion rate
FCS: Fecal clinical score
GCS: General clinical score
IAV-S: Influenza-A Virus Swine
NSP: Non-starch polysaccharide
PCV-2: Porcine Circo-Virus type 2
PRRSV: Porcine Reproductive and Respiratory Syndrome Virus
PWD: Post-weaning diarrhea
SBM: Soybean meal
